# Comment on Diala Alshiyab ‘Demographic and clinical features of rosacea in North Jordan, a 10 year university hospital retrospective study’

**DOI:** 10.1080/07853890.2025.2474174

**Published:** 2025-03-12

**Authors:** Atia Saif, Salman Hassan

**Affiliations:** ^a^ D.G.K Medical College, Dera Ghazi Khan, Pakistan; ^b^ Nishtar Medical University, Multan, Pakistan

Comment on Diala Alshiyab et al.

‘Demographic and clinical features of rosacea in North Jordan: a 10 year university hospital retrospective study’

In this issue of Annals of Medicine, Diala Alshiyab et al. demonstrate demographoc and clinical characteristics of rosacea, focusing on Fitzpatrick skin type (fair skin types) 3 and 4, also considering ethnic and racial variations in rosacea presentation. Rosacea is chronic inflammatory skin condition, causes redness on skin especially on nose and cheeks. It often appears similar to acne but unlike acne, it first starts in middle age. Skin findings include erythema, telangiectasia, papules and pustules. No comedones are present. Symptoms are aggravated by sun exposure, stress, spicy foods etc. and can be relieved by avoiding alcohol and hot beverages.

Retrospective cohort study from 2013 to 2023 by Diala Alshiyab et al. conducted at King Abdullah hospital, Jordan shows 610 patients of rosacea out of total 40,300 individuals, yielding rosacea prevalence of approximately 1.5% within hospital catchment area, with a 95% CI ranging from 1.4% to 1.6%.61% diagnoses occured in final 3 years showing upward trend. Additional details like sun exposure, occupation, stress, smoking, acne history and previous steroid use were also considered. Statistical evaluations were performed using Jamovi softsware. Authors’ study showed, 89.34% population resided in Irbid . Their cohort was chiefly female, 84.4% (mean age 44) and 15.6% were males (mean age 50). 89.8% patients had indoor employment and 17.9% were smokers. Fitzpatrick skin type 3 represented 56.4% and type 4 respresented 34.8% of patients. Erythemotelangiectatic rosacea accounted for 73.6%, followed by papulopustular rosacea at 23.4% and phymatpus rosocea 3% (significantly prevalent among males). Sun exposure acted as exacerbating factor (99.8%), temperature changes (96.1%), and history of acne in 39.3% patients. Furthermore male patients with rosacea are at elevated risk of migraine. Frontline topical medications like brimonidine, ivermectin and, metronidazole and azeliac acid are essential for controlling symptoms.

One thing, we would like to point is, there should be more research on late onset of rosacea, and phymatous rosacea prevalence in males, despite, their increased exposure to sun. This study should also have pointed importance of eye involvement, addiction history, psychological disturbances caused by rosacea, and history of use of sun screens. According to Amal Omer et al. study [1], clinical presentation of rosacea in dark skinned people is same as fair skinned.40% patients were of skin type 4, 18% of type 5 and 42% of type 6. According to article of American academy of dermatology association [2], signs of rosacea affecting eyes included, swollen eyelids, burning sensation and sensitivity. Study by Li et al. [3], shows increasing pack years of smoking was associated with elevated risk of rosacea among past smokers and with decreased risk of rosacea among current smokers. Research by Blount et al. [4] stated, link of mental health and rosacea. People with rosacea may feel less self esteem and may often be viewed as alcohol abusers. Article posted in National Rosacea Society [5] showed, 88% of patients with severe symptoms said the disorder had negatively affected their interactions with their workplace. Article by Haiyan Chen et al. [6] stated, erythema telangiectasia rosacea is most common type in female acne patients. Rational daily use of skin care products can reduce incidence of rosacea in acne patients. According to Hanlin Zhang et al. [7], Patients with rosacea should be encouraged for gentle skin care, moisturizing and use of sun screens.

I hope these additional considerations would help in enhacing discussion over this topic.

Thank you for your consideration.

## Data Availability

Not applicable as no new data was generated.
